# Supplementary material for: The Systems Biology Research Tool: evolvable open-source software
Source: BMC Syst Biol. 2008 Jun 29;2:55. doi: 10.1186/1752-0509-2-55 (PMC2446383; doi:10.1186/1752-0509-2-55)
Supplement: Additional file 1 — SBRT Archive. An archive of the current version of the Systems Biology Research Tool. [file 1752-0509-2-55-S1.zip › sbrt-1.4.0/doc/developers_guide/api/sbrt/kernel/math/comb/comp/SingleElementUnionsV2.html]

SingleElementUnionsV2


|  |  |  |
| --- | --- | --- |
| **Overview** \| **Package** \| **Class** \| **Use** \| **Tree** \| **Deprecated** \| **Index** \| **Help** | |  |
| **PREV CLASS**   **NEXT CLASS** | **FRAMES**    **NO FRAMES**     **All Classes** |  |
| SUMMARY: NESTED \| FIELD \| CONSTR \| METHOD | DETAIL: FIELD \| CONSTR \| METHOD |  |


---


## sbrt.kernel.math.comb.comp Class SingleElementUnionsV2

```
java.lang.Object
  sbrt.kernel.math.comb.comp.SingleElementUnionsV2
```

---

``` public class SingleElementUnionsV2 extends java.lang.Object ```

This class is used to compute the single-element unions of collections of
sets. A single-element union of sets will contain (at least) one element
from each set. Take, for example, the following collection of sets:  

|  |  |  |
| --- | --- | --- |
| {a, b, c} {d} {e, f}. | | |

  
The sets of all possible single-element unions from these sets are:  

|  |  |
| --- | --- |
| {a, d, e} {a, d, f} | |
| {b, d, e} {b, d, f} | |
| {c, d, e} {c, d, f}. | |

  

**Author:**
:   This class was written and documented by Jeremiah Wright
    while in the Wagner lab.

---

**Constructor Summary**

`SingleElementUnionsV2()`


| **Method Summary** | |
| --- | --- |
| `<E> java.util.Set<java.util.Set<E>>` | `getSingletonSets(java.util.Set<E> set)`             Returns a set of singleton sets created from the provided set. |
| `<E extends java.lang.Comparable<? super E>> java.util.Set<java.util.Set<E>>` | `getUnions(java.util.Collection<java.util.Set<E>> sets)`             Returns the set of single-element unions of the sets in the provided collection. |

**Methods inherited from class java.lang.Object**

`clone, equals, finalize, getClass, hashCode, notify, notifyAll, toString, wait, wait, wait`

**Constructor Detail**

### SingleElementUnionsV2

```
public SingleElementUnionsV2()
```


**Method Detail**

### getSingletonSets

```
public <E> java.util.Set<java.util.Set<E>> getSingletonSets(java.util.Set<E> set)
```

:   Returns a set of singleton sets created from the provided set. The
    singleton sets each contain a single element from the provided set.
    This can be thought of as the single-element union of the provided
    set and the empty set.

    :   **Type Parameters:**: `E` - the element type. **Parameters:**: `set` - a single set of elements. **Returns:**: a set of singleton sets created from the provided set.

---


### getUnions

```
public <E extends java.lang.Comparable<? super E>> java.util.Set<java.util.Set<E>> getUnions(java.util.Collection<java.util.Set<E>> sets)
```

:   Returns the set of single-element unions of the sets in the provided
    collection.

    :   **Type Parameters:**: `E` - the element type. **Parameters:**: `sets` - the collection of sets. **Returns:**: the set of single-element unions in the provided collection of sets.


---


|  |  |  |
| --- | --- | --- |
| **Overview** \| **Package** \| **Class** \| **Use** \| **Tree** \| **Deprecated** \| **Index** \| **Help** | |  |
| **PREV CLASS**   **NEXT CLASS** | **FRAMES**    **NO FRAMES**     **All Classes** |  |
| SUMMARY: NESTED \| FIELD \| CONSTR \| METHOD | DETAIL: FIELD \| CONSTR \| METHOD |  |


---
